# Supplementary material for: Evidence from a long-term experiment that collective risks change social norms and promote cooperation
Source: Nat Commun. 2021 Sep 15;12:5452. doi: 10.1038/s41467-021-25734-w (PMC8443614; doi:10.1038/s41467-021-25734-w)
Supplement: Supplementary file 1 — Supplementary Information [file 41467_2021_25734_MOESM1_ESM.pdf]

Evidence from a long-term experiment that collective risks change social norms and promote cooperation

Supplementary Information

Authors: Aron Szekely[1,2,3,*,†], Francesca Lipari[4,†], Alberto Antonioni[4], Mario Paolucci[1,5], Angel Sánchez[4,6,7,8], Luca Tummolini[1], Giulia Andrighetto[1,2,9]

[1] Institute of Cognitive Sciences and Technologies, Italian National Research Council, Rome, Italy
[2] Institute for Futures Studies, Stockholm, Sweden
[3] Collegio Carlo Alberto, Turin, Italy
[4] Grupo Interdisciplinar de Sistemas Complejos (GISC), Departamento de Matemáticas, Universidad Carlos III de Madrid, Leganés, Spain
[5] Institute for Research on Population and Social Policies, Italian, National Research Council Rome, Italy
[6] Instituto de Biocomputación y Física de Sistemas Complejos (BIFI), Universidad de Zaragoza, Zaragoza, Spain
[7] Unidad Mixta Interdisciplinar de Comportamiento y Complejidad Social (UMICCS), UC3M-UV-UZ, Leganés, Spain
[8] UC3M-Santander Big Data Institute (IBiDat), Universidad Carlos III de Madrid, Getafe, Spain
[9] Malardalens University, Vasteras, Sweden

* Corresponding author: aron.szekely@carloalberto.org
† These authors contributed equally

Table of Contents

Supplementary Methods *3*
- **Section 1: Experiment instructions** 3
- **Section 2: Summary statistics** 12
- **Section 3: Regressions** 12
- **Section 4: Social norm beliefs elicitation method** 14
- **Section 5: Social norms strength definition and measurement** 15
  - *Consistency* 16
  - *Accuracy* 16
  - *Specificity* 17
- **Section 6: Analysis of behavioral typology** 18

Tables and Figures *20*
- **Supplementary Table 1. Summary statistics.** 20
- **Supplementary Note 1** 20
- **Supplementary Table 2. Dropout by end of the collective-risk social dilemma** 21
- **Supplementary Note 2** 21
- **Supplementary Table 3. Predictors of contribution.** 22
- **Supplementary Table 4. Contribution according to manipulated expectations.** 23

Supplementary Table 5. Punishing points allocated by collective risk probability and treatment. **24**

Supplementary Table 6. Beliefs about punishing points by collective risk probability and treatment. **25**

Supplementary Table 7. Individual predictors of Empirical Expectations Influence and Normative Expectations Influence. **26**

Supplementary Table 8. Social norm strength according to collective risk probability **27**

Supplementary Table 9. Contribution by treatment and collective risk probability. **28**

Supplementary Table 10. Group reaching threshold by treatment and collective risk probability. **29**

Supplementary Table 11. Groups reaching the threshold according to norm strength and collective risk. **30**

Supplementary Table 12. Comparison test of cooperation level for round 15 among treatments **31**

Supplementary Note 3 .... **31**

Supplementary Table 13. Dynamics of cooperation after the change in risk by treatment32

Supplementary Note 4 .... **32**

Supplementary Table-14. Individual predictors of round 14 to 15 contribution change. .. **33**

Supplementary Fig. 1. Conditional contribution according to empirical expectations (EE) and normative expectations (NE). **34**

Supplementary Fig. 2. Behavioral types by treatments for Type 1. .... **35**

Supplementary Note 5 .... **35**

Supplementary Fig. 3. Behavioral types by treatments for Type 2. .... **36**

Supplementary Fig. 4. Social norm strength components for round and broken down by treatment. **37**

Supplementary Fig. 5. Contributions and expectations by round according to treatment. 38

Supplementary Fig. 6. Proportion of groups reaching threshold according to social norm strength. **39**

Supplementary References ***40***

Supplementary Methods

Section 1: Experiment instructions

In this section we include the translation from Spanish of the exact instructions form that participants received for the two experimental treatments, named High Low (HL) and Low High (LH). Instructions related to HL treatments only are written in **red** color while those for LH treatments only are written in **blue** color. A resumed version of the instructions was always available to participants during the entire experiment. The Spanish version of the instructions can be made available upon request. The cartoon images show on pp. 7, 8 were included in the instructions for subjects. The instructions for the Big Five, Risk preference elicitation, Autism spectrum measurement, demographic questionnaire, and Social Value Orientation are not reported here and can be requested from the authors. [Comments about experimental protocol that were not shown to participants will be shown below in brackets.]

Welcome page

Welcome and thanks for participating in this experiment. The experiment consists in seven sections. You will begin by doing the first five sections, which will take approximately 20 minutes. You can do it right after reading these instructions. These sections are mandatory and you will be automatically excluded if you will not complete them. You have until tomorrow at **10 AM (Madrid time)** to complete these first five sections. After that, you will be moved to the sixth section. You can take the first decision during this section tomorrow. Then, you will be participating in a round of the sixth section all the other days. You will have until **10 AM (Madrid time)** of the following day to make your decisions for that day. Your decisions during this section will not take more than few minutes per day. The last section, the seventh, will take few minutes to be completed.

Important rules

- Your participation is voluntary and you can leave the experiment at any time. However, in that case, you will not receive any payment.
- You are asked to not communicate with other participants and, in general, to make your decision in an independent manner. You may not share your participation link with anyone.
- Your answers will be kept confidential.
- During the entire experiment there will be not any form of deception.

Payment

You will be paid at the end of the experiment. Your payment will be the sum of all your earnings during each section. For each **30 points you gain you receive 1 EUR**, rounded to the closest integer number. Your earnings will depend on your decisions and the decisions of other participants, and partially from some randomness. Your payment will be made through PayPal, in a manner that other participants will be not able to know how much you gained. Additional instructions on how you can make extra points will follow.

If you do not complete the decisions of today or some decisions during the sixth section, you will be **automatically and permanently** excluded from the experiment. **If you will be excluded, you will not be paid**.

At the end of the experiment, one participant among those who finish the experiment will be randomly selected to gain an extra bonus. The bonus means that all the earnings of the selected participant will be multiplied by 10. We guarantee that **the randomly selected person will gain at least 100 EUR and up to 200 EUR**.

Additional information
Please remember that once you click on the "Next" button below you cannot come back to this screen. Please always read carefully all experimental instructions. We will always show you a compact version of the instructions. If you have any doubt during the experiment, please get in touch with us (cnr.ibsen@gmail.com). Please click on the "Next" button.

[After this page participants perform Big 5 (section 1), SVO test (section 2), Autism spectrum (section 3), demographic questions (section 4) and risk preference test (section 5)]

End of day 1 page
You have completed all decisions of today. Come back tomorrow at **10 AM (Madrid time)**.

Instructions of section 6 page
You are going to interact with other **5 participants**. Their identities will remain unknown for the entire experiment. At each round, you will receive a round endowment of **100 points** and you can choose how many points you want to invest in a common pot.

If your group cumulates **300 points** in the common pot all of you will save the points you did not invest.

If the cumulated investments do not reach the threshold, the system will choose a random number between 1 and 10. If this number is less than or equal to **9** (HL treatment) **6** (LH treatment) **you all will lose all points** and you will not gain any point from that round.

On the other hand, if this number is larger than **9** (HL treatment) **6** (LH treatment) all of you will save the points you did not invest.

In other words, if the points in the common pot during a round are less than 300, there is a probability of **90%** (HL treatment) **60%** (LH treatment) that you lose all points for that round.
To summarize, there are three possible outcomes for each round. If your group cumulates:

- **at least 300 points**, all participants in the group **save the points they did not invest**.
- **less than 300 points** and the system draws a number less than or equal to **9** (HL treatment) **6** (LH treatment), all participants in the group **lose all the points for that round**.
- **less than 300 points** and the system draws a number larger than **9** (HL treatment) **6** (LH treatment), all participants in the group **save the points they did not invest**.

You will take part in 28 rounds of this section of the experiment. At the beginning of each round, you will be randomly grouped with other 5 participants in the experiment.

At the end of the experiment the system will randomly select four rounds. One for each week of the experiment. The amount you have gained during those four rounds will be added to your final payment. Each round is independent from each other in terms of your earnings.

During these rounds there will be some additional questions that will allow you to gain extra points. In some rounds you will be asked to make your decisions in a different framework, so please pay attention to all the instructions. Every change will be underlined during the experiment.

At the end of the experiment, a participant will be randomly selected. The points gained by the selected person will be multiplied by 10. This means that **the selected person can gain up to 200 EUR (with a guaranteed gained of 100 EUR)**.

Important note: if you do not take a decision for more than 3 days you will be automatically excluded from the experiment and you will not gain anything from it. **If a participant does not take a decision during a round, a random decision chosen from another person in the same group will be automatically implemented.**

These instructions will be available during the entire experiment at the end of each screen.

Examples page

Example 1
In the first round you have been grouped with other 5 people. Each participant receives 100 points. You contributed 0 points and the others contribute 50 points each. As a consequence, the total amount invested in the common pot is 250 points (= 0 points + 5 x 50 points), which does not reach the needed 300 points.

The system draws a random number from 1 and 10. The number is 2, this means that all of you gain no points from this round.

Example 2
In the second round you have been grouped with other 5 people. Each participant receives 100 points. You contributed 75 points and the others contribute 75 points each. As a consequence, the total amount invested in the common pot is 450 points (= 75 points + 5 x 75 points), which does reach the needed 300 points.

As a consequence, you gain 25 points from the saved points you did not contribute (= 100 points - 75 points), as your groupmates.

Example 3
In the third round you have been grouped with other 5 people. Each participant receives 100 points. As in the first round, you contributed 0 points and the others contribute 50 points each. As a consequence, the total amount invested in the common pot is 250 points (= 0 points + 5 x 50 points), which does not reach the needed 300 points.

The system draws a random number from 1 and 10. The number is 10, this means that all of you gain the points you did not contribute. In this case, you gain from this round 100 points.

Questions page
Please answer the questions below. They are designed to help you better understand section 6. Your answers will not have any consequences for your payments. WE remind you that you can see the complete instructions for this section in a box at the bottom of this page (move the page up if necessary).

Question 1
Your group contributed in total 350 points in this round. What does it happen?
- The group contributed enough points and all group members gain saved money.
- All group members lose all points.
- All group members lose all point with probability of **90%** (HL treatment) **60%** (LH treatment).

Question 2
Your group contributed in total 150 points in this round. What does it happen?
- The group contributed enough points and all group members gain saved money.
- All group members lose all points.
- All group members lose all point with probability of **90%** (HL treatment) **60%** (LH treatment).

Question 3
You contribute 50 points in this round, all your group members contributed 250 points. How much do you gain in this round?
- 0
- 50
- 70

Question 4
You participate in this experiment always with the same 5 people in each round. [True/False]

Question 5
What does it happen if you miss three or more decisions during the experiment?
- Nothing.
- You will be excluded and not paid.

Answers page
You have correctly answered to *x* out of 5 questions. Here they follow correct answers. Section 6 will begin after this page. [Participants are shown correct answers.]

First round page (days 2-29)
You are going to begin the round X out of 28. This means that you are playing the day (X+1) out of the experiment. **You have been randomly grouped with other 5 people.**

Personal Normative Belief page (days 2-29)
How many points should a person in your group, including yourself, contribute?

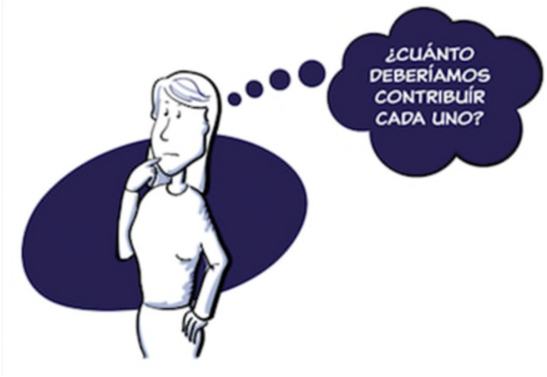

Empirical Expectations page (days 2-29)
You now have the opportunity to earn additional points. At the end of the experiment, you will be told if you have earned these points.

How many points will the other 5 people in your group spend?

Use the boxes below to indicate **the contributions you think the other people in your group will make**. Put the highest value in the box at the top and then rank the contributions in descending order. You can enter the same value for several people. In that case the order for those people does not matter. We will rank the contribution of the other people in your group in this round and compare each of them with your answers. For each answer you believe to be completely correct you will get 5 points. This means you can earn a maximum of 25 points. The less accurate your answer is, the less points you will receive. If your answer differs from the actual values by more than 5 points then you receive 0 points for those answers.

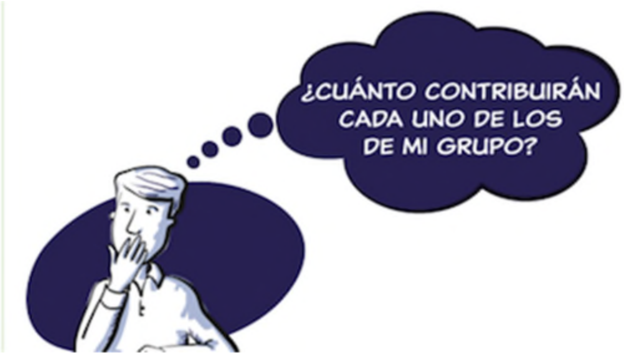

Normative Expectations page (days 2-29)
You now have the opportunity to earn additional points. At the end of the experiment, you will be told if you have earned these points.

How many points will the other 5 people in your group think you all should spend?

Use the boxes below to indicate **how many points each of the people in your group think each of you should contribute**. Put the highest value in the box at the top and then rank the contributions in descending order. You can enter the same value for several people. In that case the order for those people does not matter. We will rank the responses to the previous question ('How many points should a person in your group, including yourself, contribute?') of the other people in your group in this round and compare each of them to your responses. For each answer you believe to be completely correct you will get 5 points. This means that you can earn a maximum of 25 points. The less accurate your answer is, the less points you will receive. If your answer differs from the actual values by more than 5 points, then you receive 0 points for these answers.

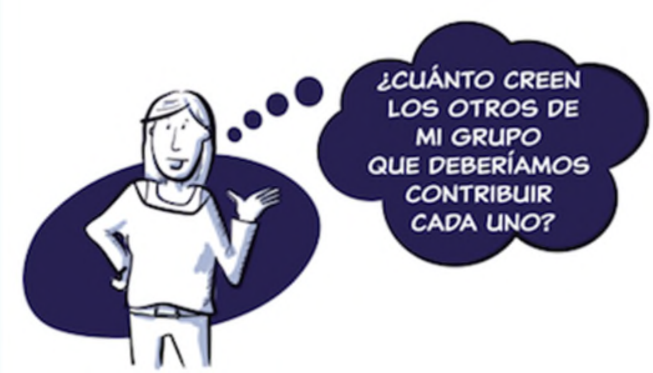

Contribution page (days 2-29)
How many points do you want to invest?

Conditional scenario page
[for days 2/5/8/9/12/15/16/19/22/23/26/29 only]
In this round, we ask you to make an additional decision to earn extra points. We ask you what you would contribute in different scenarios. For each of the four situations presented, indicate what you would do. Since we will record the responses of each group member as well as what they believe, we will implement your choice to the case that it actually happened.

Specifically, you have all indicated to us how much you think should be invested. From here, we select which of your decisions are carried out. For example, if the majority of your group members put in at least 50 points and believe that you all should spend at least 50 points, then we will use the answer you have given us in the first row to calculate the group's contribution for this additional round.

How many points will you invest if:

1. the majority of your group members put in **at least 50 points** and believe that you all should spend **at least 50 points**.
2. the majority of your group members put in **less than 50 points** and believe that you all should spend **at least 50 points**.
3. the majority of your group members put in **at least 50 points** and believe that you all should spend **less than 50 points**.
4. the majority of your group members put in **less than 50 points** and believe that you all should spend **less than 50 points**.

End of the round page (days 2-29)
You have completed all decisions of today. Come back tomorrow at **10 AM (Madrid time)**.

Results of the previous round page (days 2-29)
The previous round has finished. You have begun with **100 points**. **You invested XX points and your group contributed a total of YY points**. The complete list of contributions follows below. Contributions are listed in a random order.

[Option 1: Threshold achieved]

This means that your group accumulated enough points, and you all save the points you did not spend in this round.

[Option 2: Threshold not achieved, and points saved]

This means that your group did not accumulate enough points. The system randomly took the number 10 and you all saved the points you did not spend in this round.

[Option 3: Threshold not achieved, and points lost]

This means that your group did not accumulate enough points. The system randomly took the number 1 and you all loose the points you did not spend in this round.

Results of the previous round page: conditional scenarios
(days 3/6/9/10/13/16/17/20/23/24/27/30 only)
You have begun with **100 points**. **You invested XX points and your group contributed a total of YY points**. The complete list of contributions is the same of the above one.
[Note that the random number for conditional scenarios is not necessarily the same]

[Option 1: Threshold achieved]

This means that your group accumulated enough points, and you all save the points you did not spend in this round.

[Option 2: Threshold not achieved, and points saved]

This means that your group did not accumulate enough points. The system randomly took the number 10 and you all saved the points you did not spend in this round.

[Option 3: Threshold not achieved, and points lost]

This means that your group did not accumulate enough points. The system randomly took the number 1 and you all loose the points you did not spend in this round.

Your total earnings in this round are ZZ points.

15th round page (also shown in following 3 rounds)
There is a change in the experiment. From this round until the end of the experiment, there is a change. **The probability that you all lose all your points if the common pot does not reach 300 points is now of 60%** (LH treatment) **90%** (HL treatment). All other settings remain the same as before. As before, if your group does not reach the needed points the system will randomly draw a number from 1 to 10 and if this number is equal to or less than **6** (LH treatment), **9** (HL treatment). If this number is larger than **6** (LH treatment), **9** (HL treatment) you keep all saved points.

After 28th round
You have been paired with another randomly chosen participant. You will not know the identity of the other person and the other person will not know yours.

All participants, including yourself, receive 30 points. You are now asked to take decisions in these hypothetical situations that will be applied to the person with whom you are paired and according to the level of contribution in the last round that person made. You can use up to ten points in this round. Each point that you use will decrease the amount of points of the other person by three. For instance, if you use 5 points you will remove 15 points from the other person.

The other person will also have to decide how many points wants to use to decrease your amount of points according to what you contributed in the last round.

At the end of the experiment, the system will randomly implement one of your decisions or one of those of the other participant. If you don't take a decision in this phase, you want gain anything from this part of the experiment.

How many points you want to use to decrease other person's amount if that person:

- invested less than 50 points in the 28th round?
- invested 50 points in the 28th round?
- invested more than 50 points in the 28th round?

After punishment decision
Now we ask you to think how many points the other person used in the previous question. Specifically, we ask you how much do you think the other persons in the experiment used to decrease the gains of their paired participant. Remember that all of you received 30 points and have been asked to use some of them to reduce the gains of their paired participant as a function of what they did in the 28th round.

Each of your answers will be compared with the average answers from all participants in this experiment and if you will estimate this quantity correctly enough you will gain 10 additional points. You can gain these points for each correct answer. The closer your answer is to the average the more you gain. If you don't take a decision in this phase, you want gain anything from this part of the experiment.

How many points do you think that the other participants in this experiment used to decrease the gain of their paired participant if:

- the other paired participant invested less than 50 points in the 28th round?
- the other paired participant invested 50 points in the 28th round?
- the other paired participant invested more than 50 points in the 28th round?

Section 2: Summary statistics

The basic statistics of our participants are shown in Supplementary Table 1 while the drop-out rate and characteristics of those who dropped out are presented in Supplementary Table 2.

Section 3: Regressions

In this section we present details on the different regression models on our data and that we discuss in text. Statistical significance is calculated using two-sided *t*-tests for all analyses except in Supplementary Table 10 which uses two-sided *z*-tests.

There are three roles for subjects in our experiment: active, inactive, and excluded. We call a subject active when he or she makes a decision in a round and a subject as inactive when he or she does not make a decision in a round but is not excluded and still has the possibility of making decisions later in the experiment. Excluded subjects are those who (*i*) either did not complete the start of experiment questionnaires or (*ii*) missed 3 or more decisions during the experiment.

Subjects can be inactive in a round for multiple reasons. They may, for instance, take none of their decisions or take a subset of them. In the latter case, we still have some information about their decisions. Whenever we remove observations due to inactivity, we do so only on those variables for which they did not respond but keep the information that is available.

In Supplementary Table 3, we fit a series of linear regression models testing the variables that predict contribution. Standard errors are clustered according to subject id. We operationalize empirical expectations by calculating the mean empirical expectations for each individual in each round. We operationalize normative expectations in the same way. Subjects who are inactive in terms of contribution are removed from the analysis as are those who are excluded.

We find that empirical expectations, normative expectations, and personal normative beliefs are consistent and strong predictors of contribution (models 1-4). The associations are, for every specification, substantive and they remain so even when extensive controls are added (models 3 and 4). Apart from these variables, prosocial preferences are consistently associated positively with contributions. Risk preferences are marginally significantly associated with contribution such that more risk seeking people are predicted to contribute less, but this is only the case in one model (Model 3) but not in the other (Model 4). Neither the Autism Spectrum Quotient nor the Big Five personality traits predict contribution. Among the additional control variables, we find that females contribute somewhat more than males (Model 4: b=2.163, s.e.=1.025, p=0.036).

Supplementary Table 4 shows the results from linear regression analyses that test the association between conditional contributions and empirical and normative expectations. Standard errors clustered according to subject id. This analysis shows that subjects change their behavior both according to empirical and normative expectations.

In the experiment, subjects' who did not actively make a decision in the conditional contribution section contributed the same amount to the different categories as a randomly selected active subject from their group. These observations are excluded from the analysis.

Supplementary Table 5 tests the differences in punishment points spending by collective risk probability (Model 1) and by collective risk probability and treatment (Model 2). This is done using linear regressions with cluster robust standard errors at the individual level. Only active subjects are included in the analysis.

Supplementary Table 6 tests the differences in *beliefs* about punishment points spending of others by collective risk probability (Model 1) and by collective risk probability and treatment (Model 2). Subjects anticipate difference in punishing strength according to risk. They anticipate greater punishing of non-contributors in the Low High treatment at 7.52, 95% CI [6.93, 8.11] than in the High Low treatment 6.64, 95% CI [6.05, 7.23] (difference: p=0.039). Also, they do not differ in their expectations about punishment of 50 contributors at 3.14, 95% CI [2.50, 3.77] and 3.56, 95% CI [2.95, 4.16] or more than 50 contributors at 2.64, 95% CI [1.97, 3.30] and 2.96, 95% CI [2.32, 3.61]. This is done using linear regressions with cluster robust standard errors at the individual level. Only active subjects are included in the analysis.

Supplementary Table 7 shows the results of linear regression models testing the individual predictors of Empirical Expectations Influence and Normative Expectations Influence.

In Supplementary Table 8 we test the strength of social norms (see Section 5 for details) using a linear regression. Each observation represents the social norm strength of one group in a round. We exclude groups in which three or more participants were inactive for any of the relevant responses (contributions or expectations). Moreover, we only consider social norm strength from round 3 onwards as they need time for the dynamics to differ. Including earlier rounds does not change these results.

In Supplementary Table 9 we test contributions by subjects according to collective risk probability (Model 1) and then by both collective risk probability and treatment (Model 2). We do so by using linear regressions with clustered standard errors according to individual. Subjects who are inactive in terms of contribution are removed from the analysis as are those who are excluded.

In Supplementary Table 10, we test the odds using logistic regressions that a group reaches the threshold (300 points) or not. We do this first by collective risk probability (Model 1) and then by both collective risk probability and treatment (Model 2). To avoid penalizing groups who had inactive subjects, in the experiment, inactive subjects contributed the same amount as a randomly selected active subject from their group. This may have implications for these results. In order to account for this, we exclude from the analysis all groups in which three or more group-members did not make an active contribution decision.

In Supplementary Table 11 (also plotted in Supplementary Fig. 6) we test whether groups with stronger social norms have a higher probability of reaching the threshold. We use one observation per group. We exclude groups in which three or more participants were inactive for any of the relevant responses (contributions or expectations).

In Supplementary Tables 12 and 13 we test Hypothesis 4. Throughout these analyses, we use round 14 contributions in the two treatments to provide baseline contribution levels under low and high risk. We then ask, which treatment—High Low or Low High, reaches the baseline contribution

level quicker following the change in risk. We use this baseline to provide an empirical level to which High Low and Low High should reach in round 15 if there is no stability in contributions. In both analyses we use linear regressions with cluster robust standard errors according to subject id. Contributions are only included for active subjects.

Section 4: Social norm beliefs elicitation method

During our experiment, we collect a set of expectations to assess the presence of a social norm, its endorsement and its causal effect on actual behavior.

According to Bicchieri[1], people's minds are characterized by a web of beliefs that motivates their behaviors. Some of those beliefs are personal and defined by people's attitudes or creeds, some others are called social expectations because they are based on what others do. Within the first set of beliefs the Personal Normative Beliefs are those about which actions the person thinks should be done in specific circumstance. We associate such beliefs to the so called first-order beliefs. On the other hand, social beliefs are divided in two categories. We have empirical expectations that express people's expectations concerning what *others* do in specific circumstance, and normative expectations that are beliefs about what *others* think should be done. The normative expectations are second order beliefs- beliefs about what others believe, that is, "beliefs about beliefs" and sometimes they are also accompanied by a certain willingness to punish deviants.

A social norm is a collective practice sustained by empirical and normative expectations and by preferences conditional on both these expectations. Bicchieri's definition, hence, implies that the two expectations need to be mutually active and elicited when assessing the presence of a social norm. If empirical and normative expectations do not mutually inform the behavior of people, then no social norm is present but other norms could be in place (i.e. descriptive norms, customs, morals).

The elicitation methods applied in the manuscript considers such requirement. Moreover, in order to ensure truth-telling the elicitation questions are incentivized such that participants received ten additional points for each exact answer (and less points the more their answers were inaccurate). We elicited expectations in an ordered way *and* we incentivised them based on this ordering. That is, subjects knew that we would compare their ordered list of responses (five items) to the ordered list of true values for their group (empirical expectations to contributions and personal normative beliefs to normative expectations) and we would compare the two to calculate how much they earned. The closer they matched, the more they earned.

However, the order in which the norm-elicitation and behavioral experiments are conducted may systematically affect the elicited norms and behavior. On the one hand, eliciting norms after having elicited behavior may introduce systematic biases in the measurement of norms like self-serving biases[2,3]. On the other, if elicitation occurs before the behavior, people may focus their attention on the norms that prevail in that situation, and may thus affect behaviour focus their attention on the norms that prevail in that situation, and may thus affect behaviour, and in doing so they incur in what is called situational cues biases[4–7]. To avoid those biases, we elicited people's beliefs sometimes before and sometimes after their behavioral choice with the order of beliefs elicitation was randomly selected.

The presence of social norm and its endorsement are not the same phenomenon. In fact, the endorsement of a social norm implies that people, following the norm, are willing to punish the deviants. Hence, people's willingness to punish needs to be elicited along with the expectations of others' willingness to punish. Yet, given that the paper aims to study the emergence and presence of a social norm, rather than its endorsement, the elicitation of both people's punishment and people's expectations on others' punishment has been done only at the last round of the game (30th day).

Finally, the emergence and presence of a social norm might affect people's in-game behavior. Yet, to study the causal connection between the presence of a social norm and people behavior we need to measure how much people are willing to change their behavior if that social norm were to change. To do so, we created a conditional contribution scenario in which people were asked to contribute conditional on if the majority of their group members put in [*at least 50 points/less than 50 points*] and believe that you should all spend [*at least 50 points/less than 50 points*]. We incentivized this by identifying the EE and NE combination that held in the subjects' group and they were additionally paid for this. This elicitation took place in rounds 1, 5, 10, 14, 15, 19, 24, and 28 and was used for the causal analysis and the base for building the analysis of behavioral typology.

Section 5: Social norms strength definition and measurement

All three measures (consistency, accuracy, and specificity) are necessary for a social norm to exist. If expectations are not consistent nor accurate, then, respectively, there is no coordination in expectations and expectations do not reflect reality. In both cases coordinated behavior is precluded. While if a norm is unrestricted in terms of the socially approved action, then it is meaningless. Our three measures are calculated considering the ordered ranking of all the set of individual's expectations within the groups and across treatment. For each participant, p, in group G_p that contains other m individuals, playing at time t we rank, (R is the rank order), participant's expectations and contributions. Further, we define G_p^{-p} as the group G_p excluding participant p, that contains *(m-1)* individuals. For example, $EE_R^p(t)$ is the ranked list of all the EE of participant p playing at time *t*. The ranked ordered allow us to have a granular vision of individuals' expectations.

Consider the following example, which uses the measure of specificity, to illustrate why it is essential to use distributions instead of only averages. If we were to use each individual's average responses the measure would conflate situations in which people believe there are two (or more) norms within a group and those that believe there is a single norm. Imagine that a group's average empirical expectation is 50. Individual *i* in that group reports her empirical expectations as (0, 0, 50, 100, 100) while individual *j*, also in that group, thinks that the distribution is (50, 50, 50, 50, 50). If we use the averaging approach, then both individuals receive a score of 0 and are thus interchangeable (since the group average is 50 and the average expectation for each is also 50). Conversely, with our approach *i* receives a score of 200 (|0-50| + |0-50| + |50-50| + |100-50| + |100-50|) while *j* receives a score of 0 (50-50, 50-50, 50-50, 50-50, 50-50) allowing us to separate between the radically different situations.

Consistency

Consistency may be defined as the difference among individual's expectations and group's expectations that are both ordered by the rank (Eq. (1)). $EE_R^p(t)$ and $NE_R^p(t)$ are the ordered empirical and normative expectations of participant *p* in group *g* at time *t*. Since individuals do not know the identity of the other people in their group, we can order their expectations and compare their orderings with the others in their group. In fact, $EE_R^q(t)$ and $NE_R^q(t)$ are the ordered empirical and normative expectations of individuals belonging to the group G_p^{-p} at time *t*. We calculate consistency of the entire population at time *t* in the following way:

$$Consistency(t) = 1 - \frac{\sum_p \sum_{q \in G_p^{-p}} \sum_{R \in [1,m-1]} \left(\left| EE_R^p(t) - EE_R^q(t) \right| + \left| NE_R^p(t) - NE_R^q(t) \right| \right)}{max \left\{ \sum_p \sum_{q \in G_p^{-p}} \sum_{R \in [1,m-1]} \left(\left| EE_R^p(t) - EE_R^q(t) \right| + \left| NE_R^p(t) - NE_R^q(t) \right| \right) \right\}} \quad (1)$$

As the deviation between participant *p*'s ranked EE (NE) and his group mates' ranked EE (NE). For example, let us imagine that participant *p* has the following ordered EE (99, 80, 70, 40 ,0), that is he expects that at least one of his group mates will contribute 99, another 80, another 70 and so forth, he, of course, does not know who will contribute 99 or 80 or 70 etc. Now imagine that one of his group mates has the following ordered EE list (90, 72, 50, 35, 20). We calculate the deviance between the two lists for each rank, for example for the first rank the deviance will be of 9 points (|90-99|=9), and so forth with the rest of the ranks and participants.

Consistency measures how far participants' expectations, about how many other participants are like-minded, i.e. they are different from one to another.

The smaller the EE and NE deviance (i.e. the deviance is closed to 0) among participants, the higher is the match of expectations within each group. When consistency is high, i.e. near to 1, the distributions of expectations within each group match. When consistency is low, hence equal to 0, the deviances among expectations is maximal and there is no common knowledge within the group about how the others will and should behave. All participants have different expectations. When consistency reach a level of nearly 0,5 we are presented with a situation in which several different beliefs coexist.

Accuracy

Through accuracy we measure whether the ranks of ordered empirical and normative expectations of an individual are close (hence accurate), respectively, to the ranks of ordered cooperation level of the other participants of the group (i.e. $C_R^{G_p^{-p}}(t)$) and the ranks of ordered personal normative beliefs of the participants in the group (i.e. $PNB_R^{G_p^{-p}}(t)$) (Eq. (2)). We calculate accuracy of the entire population at time *t* in the following way:

$$Accuracy(t) = 1 - \frac{\sum_p \sum_{R \in [1,m-1]} \left(\left| EE_R^p(t) - C_R^{G_p^{-p}}(t) \right| + \left| NE_R^p(t) - PNB_R^{G_p^{-p}}(t) \right| \right)}{max \left\{ \sum_p \sum_{R \in [1,m-1]} \left(\left| EE_R^p(t) - C_R^{G_p^{-p}}(t) \right| + \left| NE_R^p(t) - PNB_R^{G_p^{-p}}(t) \right| \right) \right\}} \quad (2)$$

For example, let us consider participant p's ordered EE (99, 80, 70, 40 ,0). Imagine that from his group mates we have their cooperation levels, for example (45, 0, 78, 23, 85) and hence we can create an ordered list of such levels, i.e. (85, 78, 45, 23,0) and compare it with the ordered EE of participant *p*. We calculate the deviance between the two, for example the difference between the highest EE and the highest contribution is of 14 point (= |99-85|), the second highest difference is of 2 points, the third difference is of 15 points and so forth: meaning that participant p overestimates the number of people that she expects will contribute. The same overshooting could happen when we compare participant's NE and the others' PNB.

Accuracy is a measure of how much participants' expectations (both empirical and normative) "forecast" what the others will do or think that is appropriate to do. Or in other words, how much the expectations are far (i.e. overshooting) or close (i.e. accuracy) from what the others will do and from what they believe is the right thing to do.

When accuracy is high, then there is no difference between what participants expect from the others' contributions and their actual contributions and there is no difference between participants' normative expectations and what the others believe is appropriate to do. A convergence towards a unique strategy on how to resolve the dilemma occurs in a faster time. When accuracy starts decreasing (i.e. less than 1), such differences, on both empirical and normative perspective, is wide hence the common strategy will need larger time to emerge. From this measure we are not able to identify the nature of the common strategy (i.e. cooperation or defection), we only know that participants will coordinate on a common strategy.

Specificity

Through specificity we measure the extent to which situations differ in the range of behavioral responses that are considered appropriate, or the extent to which the situation constrains or affords opportunities for behavioral options (Eq. (3)). This construct comes from the theoretical and empirical work[8,9]. We measure whether the individual's empirical and normative expectations are close, respectively, to the group average empirical and normative expectations. Roughly put, for individual p in group G_p, and the average of each measure is calculated at the group level.
We calculate specificity of the entire population at time *t* in the following way:

$$Specificity(t) = 1 - \frac{\sum_p \sum_{R\in[1,m-1]} \left(\left| EE_R^p(t) - \overline{EE_R^{G_p}(t)} \right| + \left| NE_R^p(t) - \overline{NE_R^{G_p}(t)} \right| \right)}{max\left\{ \sum_p \sum_{R\in[1,m-1]} \left(\left| EE_R^p(t) - \overline{EE_R^{G_p}(t)} \right| + \left| NE_R^p(t) - \overline{NE_R^{G_p}(t)} \right| \right) \right\}} \quad (3)$$

Specificity measures how much participant's expectations are far from the "average mind" of the group.

We can finally calculate norm strength in the following way:

$$norm\ strength = consistency \times accuracy \times specificty. \quad (4)$$

This way of calculating norm strength implies that all components are necessary for a norm to exists. In Figure 3 in the main text we show the social norms dynamics according to the measures aforementioned. Supplementary Fig. 4 also shows the social norm dynamics broken down into the three components.

To help with the interpretation of norm strength, we created the Random Decision Baseline. This reflects what values for social norm strength components would be observed if subjects were making completely random decisions, i.e. uniformly distributed between 0 and 100, for contribution, empirical expectations, normative expectations, and personal normative belief. This scenario represents the baseline case for the values of consistency (0.6895), accuracy (0.8133), specificity (0.7552) and social norm strength (0.4235) for all rounds and both treatments. The computed values are the average of 1000 simulated setups.

Section 6: Analysis of behavioral typology

The four conditional contributions can be summarized into two measures of responsiveness to empirical and normative expectations: Empirical Expectation Influence (EEI), which represents subjects' sensitivity to empirical expectations (Eq. (5)), and Normative Expectation Influence (NEI), which represents subjects' sensitivity to normative expectations (Eq. (6)). We consider individual conditional contributions ${C^i}_{xy}$ as the contribution of subject i, averaged across all subject's answers to conditional contributions, in the scenario in which empirical expectations are set to be x (h: high, l: low) and normative expectations to be y (h: high, l: low). The measure EEI (NEI) quantifies the change in contribution from a scenario in which empirical (normative) expectations are low to a scenario in which empirical (normative) expectations are high, while keeping normative (empirical) expectations fixed. Specifically, we have that:

$$EEI^i = \frac{\left(C^i_{hh} - C^i_{lh}\right) + \left(C^i_{hl} - C^i_{ll}\right)}{2} \tag{5}$$

and

$$NEI^i = \frac{\left(C^i_{hh} - C^i_{lh}\right) + \left(C^i_{hl} - C^i_{ll}\right)}{2} \tag{6}$$

Through these responsiveness measures we are able to identify five clusters of behaviors. The numerosity of the clusters is chosen by the k-means clustering algorithm, that groups the data in a way that it both minimizes the dispersion within clusters and maximizes the distance among centroids of different clusters. The algorithm, that does not assume beforehand any specific number of types of behaviors, finds that k = 5 clusters is the optimal number of groups according to the Davies-Bouldin index. Moreover, the number of types is also supported by theoretical assumptions in related works where similar types are defined according to qualitative procedures.

As a robustness check, instead of using absolute values for EEI and NEI, we show empirical and normative expectation influences represented in polar coordinates, i.e. an angle theta and a radius rho (Eq. (7)). Intuitively, the angle weights the influence of EE against NE (or vice versa), e.g., it is 45 degrees (or 135, 225 or 315 degrees, that is, 45 degrees in another quadrant, according to the

signs of EEI and NEI), when EEI and NEI weigh the same on a participant's decision. The angle is defined as:

$$\theta = arctan\left(\frac{NEI^i}{EEI^i}\right) \tag{7}$$

where NEI and EEI follow the definitions above. On the other hand, the radius rho is simply the intensity of all possible deviations from the low expectation case, i.e., the difference in contribution from a low EE (or NE) scenario to a high EE (or NE) scenario:

$$\rho = \frac{(|C_{hh} - C_{lh}| + |C_{hl} - C_{ll}| + |C_{hh} - C_{hl}| + |C_{lh} - C_{ll}|)}{300} \tag{8}$$

The normalization term of 300 represents the maximum possible deviation and it is used to have a normalized measure defined between 0 and 1. HH, HL, LH, LL are the corresponding mean contribution values in the four hypothetical scenarios.

We then plot in Supplementary Fig. 6 and Supplementary Fig. 7 the results of observed behavioral types for both measurements by treatments and for both treatments (aggregated):

- Type 1: according to absolute values of NEI and EEI (Supplementary Fig. 2)
- Type 2: according to polar coordinates as theta and rho (Supplementary Fig. 3)

We conclude that behavioral types emerge for both kinds of measurements and they are consistent between treatments in terms of observed proportions.

Tables and Figures

Supplementary Table 1. Summary statistics.

Variable	Round 1			Round 28		
	High-Low	*Low-High*	*Overall*	*High-Low*	*Low-High*	*Overall*
Number of subjects	148	138	286	145	118	263
Age	30.39	29.78	30.09	30.56	30.14	30.37
(years)	(11.41)	(10.54)	(10.98)	(11.46)	(10.64)	(11.08)
Female	0.59	0.53	0.56	0.60	0.55	0.58
(proportion)	(0.51)	(0.52)	(0.51)	(0.51)	(0.52)	(0.51)
Student	0.53	0.47	0.50	0.53	0.45	0.49
(proportion)	(0.50)	(0.50)	(0.50)	(0.50)	(0.50)	(0.50)
Experienced	0.48	0.37	0.43	0.48	0.39	0.44
(proportion)[a]	(0.50)	(0.48)	(0.50)	(0.50)	(0.49)	(0.50)
Political orientation	3.30	3.17	3.23	3.32	3.24	3.28
(1-7)[b]	(1.50)	(1.43)	(1.46)	(1.51)	(1.47)	(1.49)
SVO angle[c]	25.64	27.86	26.71	25.67	27.25	26.38
	(12.52)	(12.00)	(12.30)	(12.57)	(12.10)	(12.36)
Risk[d]	1.99	2.25	2.12	1.99	2.24	2.10
	(1.39)	(1.51)	(1.45)	(1.40)	(1.54)	(1.46)
ASQ[e]	18.22	17.22	17.74	18.13	17.69	17.94
	(6.25)	(5.26)	(5.81)	(6.28)	(5.36)	(5.88)
Big Five						
Extraversion	26.51	26.85	26.67	26.66	26.48	26.58
	(6.47)	(6.61)	(6.53)	(6.44)	(6.83)	(6.61)
Agreeableness	34.17	34.62	34.39	34.26	34.49	34.37
	(5.32)	(4.96)	(5.15)	(5.28)	(4.94)	(5.12)
Conscientiousness	32.39	32.67	32.52	32.41	33.09	32.72
	(6.10)	(6.18)	(6.13)	(6.15)	(6.11)	(6.13)
Neuroticism	22.88	21.86	22.38	22.81	22.26	22.57
	(6.88)	(6.88)	(6.88)	(6.92)	(6.99)	(6.94)
Openness	38.61	39.26	38.92	38.64	39.03	38.82
	(6.37)	(6.00)	(6.19)	(6.41)	(6.12)	(6.27)

Notes: Parentheses show standard deviations. [a]Experience with experiments; [b]higher score indicates more right-wing; [c]Social Value Orientation; [d]higher value indicates more risk-seeking; [e]Autism Spectrum Quotient.

Supplementary Note 1

To check whether there are differences between our samples in the High Low and the Low High treatments, we conducted a series of 13 unpaired *t*-tests comparing the samples at round 1 in the two treatments. None of the comparisons were significant at the 5% level (mean=0.351, s.d.=0.218 min.=0.060, and max.=0.691) although one, for experience with experiments, is close at p=0.060. Even if this is treated as significant, there are no systematic differences across the treatments that would be reflected in the other variables.

Supplementary Table 2. Dropout by end of the collective-risk social dilemma.

Variable	Dropout by round 28		
	High-Low	*Low-High*	*Overall*
Dropout number	3	20	23
Age	22.00	27.60	26.87
(years)	(1.73)	(9.85)	(9.37)
Female	0.00	0.40	0.35
(proportion)	(0.00)	(0.50)	(0.49)
Student	0.67	0.60	0.61
(proportion)	(0.58)	(0.50)	(0.50)
Experienced	0.67	0.25	0.30
(proportion)[a]	(0.58)	(0.44)	(0.47)
Political orientation	2.33	2.75	2.70
(1-7)[b]	(0.58)	(1.12)	(1.06)
SVO angle[c]	24.26	31.45	30.51
	(11.63)	(11.07)	(11.15)
Risk[d]	1.67	2.35	2.26
	(1.15)	(1.35)	(1.32)
ASQ[e]	22.33	14.45	15.48
	(3.06)	(3.63)	(4.43)
Big Five			
Extraversion	19.33	29.00	27.74
	(3.06)	(4.69)	(5.56)
Agreeableness	29.67	35.40	34.65
	(6.66)	(5.14)	(5.55)
Conscientiousness	31.00	30.20	30.30
	(1.73)	(6.11)	(5.71)
Neuroticism	26.00	19.45	20.30
	(3.46)	(5.77)	(5.91)
Openness	37.00	40.60	40.13
	(4.58)	(5.14)	(5.13)

Notes: Parentheses show standard deviations. [a]Experience with experiments; [b]higher score indicates more right-wing; [c]Social Value Orientation; [d]higher value indicates more risk-seeking; [e]Autism Spectrum Quotient.

Supplementary Note 2

To test whether dropout in our study was random or not, we compare the sample composition within each treatment at round 1 and round 28. If dropout is random and/or small enough not to substantially shift the sample composition, then no average differences should be found. We undertake this comparison on each of the 13 variables show in Supplementary Table 2 for both treatments using a series of unpaired *t*-tests. For instance, checking whether age differs for High-Low treatment between rounds 1 and 28 demonstrates that it does not (30.39-30.56; t=-0.130, p=0.897). The *p*-values for the 13 comparisons for High Low have mean=0.924, s.d.=0.049, min.=0.837, and max.=0.985 while the 13 comparisons for Low High have mean=0.713, s.d.=0.112, min.=0.480, and max.=0.932

Supplementary Table 3. Predictors of contribution.

	Dependent variable: Contribution			
Independent variables	**Model 1**	**Model 2**	**Model 3**	**Model 4**
Empirical expectations	0.590***	0.477***	0.479***	0.447***
	(0.105)	(0.103)	(0.101)	(0.098)
Normative expectations	0.521***	0.212**	0.214**	0.224**
	(0.116)	(0.081)	(0.079)	(0.076)
Personal normative beliefs		0.564***	0.561***	0.547***
		(0.055)	(0.053)	(0.052)
Social Value Orientation angle			0.121**	0.117**
			(0.038)	(0.040)
Risk preferences			-0.656*	-0.475
			(0.319)	(0.336)
Autism Spectrum Quotient			-0.026	-0.022
			(0.080)	(0.084)
Big Five				
Extraversion			-0.067	-0.095
			(0.083)	(0.086)
Agreeableness			0.067	0.013
			(0.078)	(0.077)
Conscientiousness			-0.026	-0.041
			(0.068)	(0.075)
Neuroticism			0.066	0.017
			(0.064)	(0.064)
Openness			0.047	0.058
			(0.059)	(0.060)
Low (0.6) to High (0.9)				-0.404
				(1.078)
High collective risk				1.915***
				(0.576)
Low (0.6) to High (0.9) # High collective risk				0.384
				(1.001)
Age				0.024
				(0.042)
Gender				
Female				2.163*
				(1.025)
Other				1.907
				(1.702)
Student				-0.076
				(0.836)
Experienced				-0.258
				(0.414)
Political orientation (1-7)				-0.117
				(0.303)
Constant	-7.324	-14.968**	-19.383*	-16.212*
	(6.613)	(5.149)	(7.802)	(7.847)
Observations	7433	7433	7433	7433

Standard errors in parentheses
Standard errors adjusted for 284 clusters according to individual. The reference treatment and collective risk category in Model 4 is High-Low low collective risk probability. * $p < 0.05$, ** $p < 0.01$, *** $p < 0.001$

Supplementary Table 4. Contribution according to manipulated expectations.

	**Dependent variable: Conditional contribution**
Independent variables	Model 1
High EE and High NE	17.829***
	(1.259)
High EE and Low NE	9.670***
	(1.140)
Low EE and High NE	14.839***
	(1.137)
Constant	33.904***
	(1.302)
Observations	8428

Standard errors in parentheses
Standard errors adjusted for 283 clusters according to individual.
* $p < 0.05$, ** $p < 0.01$, *** $p < 0.001$

Supplementary Table 5. Punishing points allocated by collective risk probability and treatment.

	Dependent variable: punishment points allocated	
Independent variables	**Model 1**	**Model 2**
Less than 50	4.162***	3.796***
	(0.310)	(0.400)
50	0.308*	0.451*
	(0.136)	(0.188)
Low (0.6) to High (0.9)		0.082
		(0.425)
Less than 50 # Low (0.6) to High (0.9)		0.806
		(0.628)
50 # Low (0.6) to High (0.9)		-0.315
		(0.271)
Constant	2.023***	1.986***
	(0.211)	(0.286)
Observations	780	780

Standard errors in parentheses
Standard errors adjusted for 260 clusters according to individual.
* $p < 0.05$, ** $p < 0.01$, *** $p < 0.001$

Supplementary Table 6. Beliefs about punishing points by collective risk probability and treatment.

	Dependent variable: Beliefs about punishment points allocated	
Independent variables	**Model 1**	**Model 2**
Less than 50	4.223***	3.676***
	(0.304)	(0.398)
50	0.550***	0.592**
	(0.156)	(0.208)
Low (0.6) to High (0.9)		-0.329
		(0.471)
Less than 50 # Low (0.6) to High (0.9)		1.205*
		(0.611)
50 # Low (0.6) to High (0.9)		-0.092
		(0.315)
Constant	2.815***	2.965***
	(0.236)	(0.328)
Observations	780	780

Standard errors in parentheses
Standard errors adjusted for 260 clusters according to individual.
* $p < 0.05$, ** $p < 0.01$, *** $p < 0.001$

Supplementary Table 7. Individual predictors of Empirical Expectations Influence and Normative Expectations Influence.

	**Dependent variable: Empirical and Normative Expectations Influence**	
Independent variables	**EEI**	**NEI**
Personal normative beliefs	-0.188**	-0.003
	(0.070)	(0.062)
Social Value Orientation angle	-0.054	0.062
	(0.099)	(0.060)
Risk preferences	0.980	0.236
	(0.786)	(0.512)
Autism Spectrum Quotient	0.126	0.147
	(0.194)	(0.157)
Big Five		
Extraversion	-0.385	0.112
	(0.218)	(0.137)
Agreeableness	0.032	0.152
	(0.241)	(0.158)
Conscientiousness	0.446*	-0.265
	(0.193)	(0.141)
Neuroticism	0.174	-0.074
	(0.179)	(0.127)
Openness	0.116	0.167
	(0.170)	(0.119)
Age	-0.086	-0.073
	(0.100)	(0.075)
Gender		
Female	-3.256	-1.491
	(2.233)	(1.598)
Other	2.154	21.923***
	(4.740)	(5.058)
Student	-3.759	-1.515
	(2.309)	(1.658)
Experienced	-0.088	0.150
	(0.671)	(0.678)
Political orientation (1-7)	-1.554*	1.143*
	(0.632)	(0.478)
Constant	10.852	2.329
	(13.344)	(9.716)
Observations	2107	2107

Standard errors in parentheses
Standard errors adjusted for 283 clusters according to individual.
* $p < 0.05$, ** $p < 0.01$, *** $p < 0.001$

Supplementary Table 8. Social norm strength according to collective risk probability.

	**Dependent variable: Social norm strength**
Independent variables	**Model 1**
High collective risk probability	0.080***
	(0.006)
Constant	0.723***
	(0.004)
Observations	1171

Standard errors in parentheses
* $p < 0.05$, ** $p < 0.01$, *** $p < 0.001$

Notes: One observation per group. Groups with 3 or more inactive/excluded subjects are excluded from the analysis. Data from round 3 onwards used.

Supplementary Table 9. Contribution by treatment and collective risk probability.

	Dependent variable: Contribution	
Independent variables	**Model 1**	**Model 2**
High collective risk probability	4.183***	5.331***
	(0.633)	(0.838)
Low (0.6) to High (0.9)		0.454
		(1.545)
High collective risk probability # Low (0.6) to High (0.9)		-2.564*
		(1.270)
Constant	47.998***	47.788***
	(0.776)	(1.107)
Observations	7441	7441

Standard errors in parentheses
Standard errors adjusted for 285 clusters according to individual.
* $p < 0.05$, ** $p < 0.01$, *** $p < 0.001$

Supplementary Table 10. Group reaching threshold by treatment and collective risk probability.

	**Dependent variable: Log odds reaching threshold**	
Independent variables	**Model 1**	**Model 2**
High collective risk probability	1.201***	1.620***
	(0.122)	(0.176)
Low (0.6) to High (0.9)		0.090
		(0.159)
Low (0.6) to High (0.9) # High collective risk probability		-0.854***
		(0.246)
Constant	-0.088	-0.130
	(0.079)	(0.109)
Observations	1267	1267

Standard errors in parentheses
* $p < 0.05$, ** $p < 0.01$, *** $p < 0.001$

Supplementary Table 11. Groups reaching the threshold according to norm strength and collective risk.

	**Dependent variable: log odds reaching the threshold**
Independent variables	**Model 1**
Norm strength	1.757***
	(0.526)
High collective risk	1.083***
	(0.127)
Constant	-1.344**
	(0.385)
Observations	1267

Standard errors in parentheses
* $p < 0.05$, ** $p < 0.01$, *** $p < 0.001$

Notes: Logistic regression used with one observation at the group-level per rounds. Groups with 3 or more inactive/excluded subjects are removed.

Supplementary Table 12. Comparison test of cooperation level for round 15 among treatments

	**Dependent variable: Relative contribution (round 14 to 15 of the other treatment)**
Independent variables	*Model 1*
High (0.9) to Low (0.6)	2.200
	(1.497)
Constant	0.368
	(1.101)
Observations	268

Standard errors in parentheses
Contribution in round 15 relative to baseline in round 14.
* $p < 0.05$, ** $p < 0.01$, *** $p < 0.001$

Supplementary Note 3

We use round 14 contributions in the two treatments to provide baseline contribution levels under low and high risk. We use these baselines to provide a reference level that, in absence of any stabilizing mechanisms (e.g. norms), contributions should achieve in round 15. That is, if people only respond to the change in incentives due to the change in risk, round 14 contributions in High Low and round 15 contributions in Low High (both under high risk) should be similar and *vice versa*. We then ask, in which treatment—High Low or Low High—does round 15 contribution get closer to the baseline contribution level?

The constant in the model indicates that contributions in round 15 of Low High were essentially the same as contributions in round 14 of High Low, and, if anything is slightly higher (52.26 vs. 51.89) (*b*=0.368, *p*=0.738, 95% CIs [-1.800, 2.537]). While the sum of the constant and High Low indicates that contributions in round 15 of High Low did not collapse to the contribution levels in round 14 of Low High (49.29 vs. 46.72) (*b*=2.568, *p*=0.012, 95% CIs [.571, 4.565]).

We next check whether the differences vary across treatments. We do this by testing the difference between 0.368 and -2.568. This is because the direction of the difference between round 15 contributions and the baseline level are opposite in the treatments. Round 15 contributions in Low High increase 0.368 more than the reference level (hence +0.368) while round 15 contributions in High Low decrease insufficiently and do not reach the reference level (hence -2.568). In other words, we capture not only the difference between points but also the "sign" (increasing vs decreasing). From the regression, this is calculated as *constant* * 2 + *High Low* (*b*=2.937, *p*=0.051, 95% CI [-.0112, 5.884]).

Supplementary Table 13. Dynamics of cooperation after the change in risk by treatment

	**Dependent variable: Relative contribution (round 15 to 28)**
Independent variables	*Model 1*
Low (0.6) to High (0.9)	-3.699**
	(1.263)
Round recode	-0.331**
	(0.100)
Low (0.6) to High (0.9) # Round recode	0.270*
	(0.112)
Constant	3.210**
	(1.092)
Observations	3662

Standard errors in parentheses
Standard errors adjusted for 268 clusters according to individual. Round has been recoded so that Round 15 is 0 and Round 28 is 14.
* $p < 0.05$, ** $p < 0.01$, *** $p < 0.001$

Supplementary Note 4

To ease interpretation, we recode round (Round recode) so that the start of part 2 has the value of 0. We find that the contributions in rounds 15-28 decrease for High Low (b=-.331, p=0.001, 95% CI [-.529, -.134]) while contributions remain stable for Low High (b=-.0614, p=0.216, 95% CI [-.159, .036]). Moreover, the slopes for round recode differ between treatment (b=.270, p=0.017, 95% CI [.050, .490]). Combined with the previous findings, this indicates that in Low High, contributions jump up to the baseline level and then stay stable while in High Low, contributions start above the baseline and slowly decline over time to match it.

Supplementary Table 14. Individual predictors of round 14 to 15 contribution change.

	**Dependent variable: Change in contribution (Round 15 contribution – round 14 contribution)**	
Independent variables	**High Low**	**Low High**
Personal normative beliefs	0.651***	0.582*
	(0.080)	(0.225)
Social Value Orientation angle	0.067	-0.095
	(0.066)	(0.154)
Risk preferences	-0.324	0.112
	(0.598)	(1.263)
Autism Spectrum Quotient	-0.377*	0.438
	(0.162)	(0.400)
Big Five		
Extraversion	-0.378*	0.048
	(0.162)	(0.339)
Agreeableness	0.013	0.593
	(0.180)	(0.415)
Conscientiousness	0.336*	0.079
	(0.150)	(0.332)
Neuroticism	0.184	0.041
	(0.132)	(0.311)
Openness	0.255	0.204
	(0.129)	(0.311)
Age	-0.123	-0.082
	(0.080)	(0.202)
Gender		
Female	2.793	-6.670
	(1.690)	(4.142)
Other	-11.393	-4.730
	(10.136)	(19.408)
Student	-1.387	-1.510
	(1.827)	(4.157)
Experienced	-0.242	-1.348
	(0.647)	(1.428)
Political orientation (1-7)	0.491	-0.398
	(0.541)	(1.303)
Constant	-44.158***	-54.766*
	(12.212)	(27.009)
Observations	145	122

Standard errors in parentheses
* $p < 0.05$, ** $p < 0.01$, *** $p < 0.001$

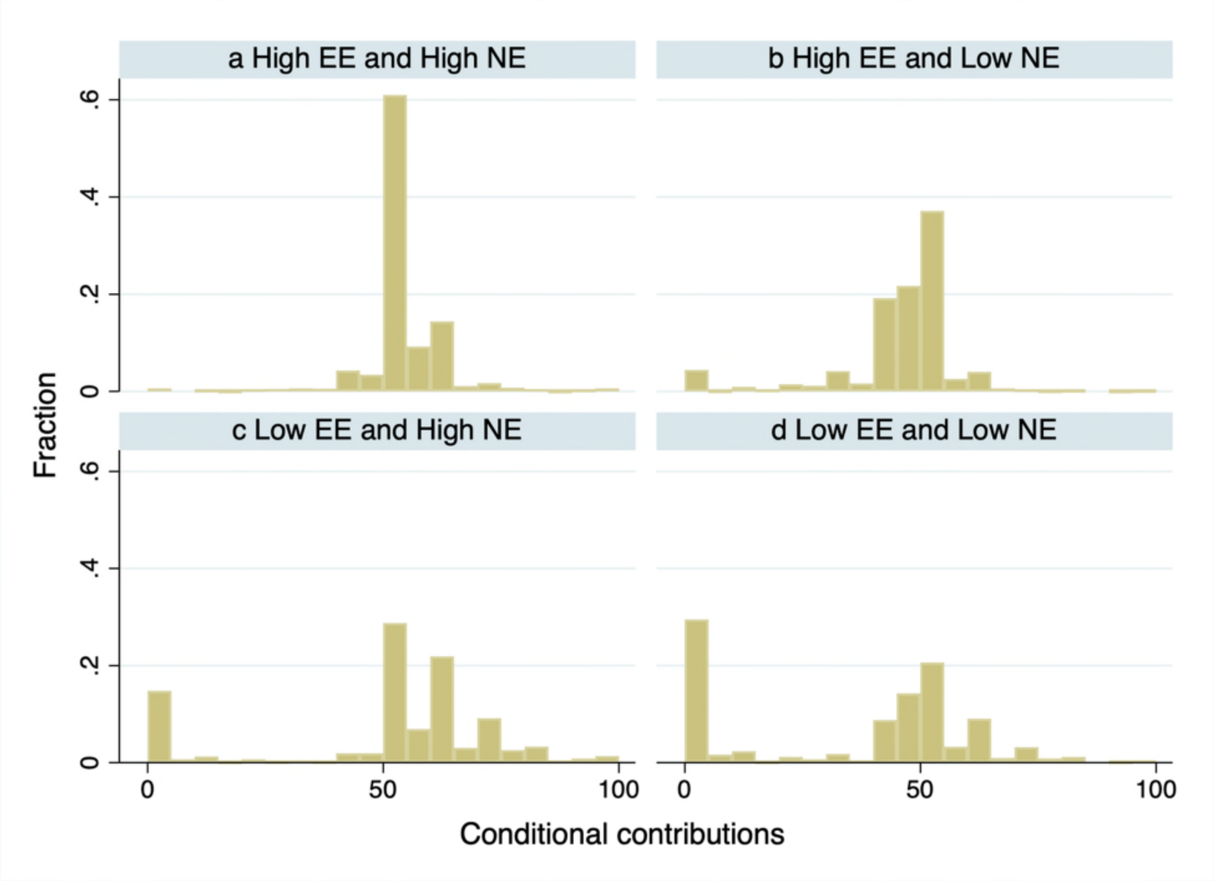

Supplementary Fig. 1. Conditional contribution according to empirical expectations (EE) and normative expectations (NE). **a** High empirical and high normative expectations. **b** High empirical and low normative expectations. **c** Low empirical and high normative expectations. **d** Low empirical and low normative expectations. High expectations implies at least 50 points and low expectations implies less than 50 points. *n*=283 individuals repeatedly measured during the experiment. Source data are provided as a Source Data file.

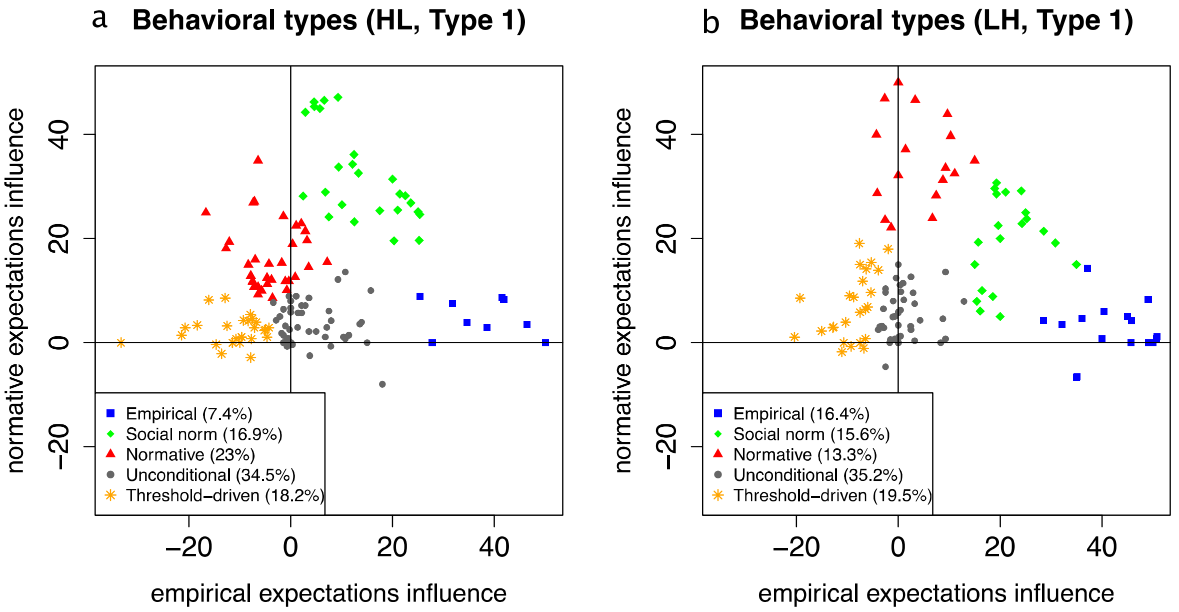

Supplementary Fig. 2. Behavioral types by treatments for Type 1. a empirical and normative expectations influence in the High Low treatment. **b** empirical and normative expectations influence in the High Low treatment. Source data are provided as a Source Data file.

Supplementary Note 5

Using the Type 1 classification, we find the following distribution of types across treatment. In High Low, there are 7% (11/148) Empirical, 17% (25/148) Social norm, 23% (34/148) Normative, 35% (51/148) Unconditional, and 18% (27/148) Threshold driven types. And, in Low High, there are 16% (21/128) Empirical, 16% (20/128) Social norm, 13% (17/128) Normative, 35% (45/128) Unconditional, and 20% (25/128) Threshold driven types.

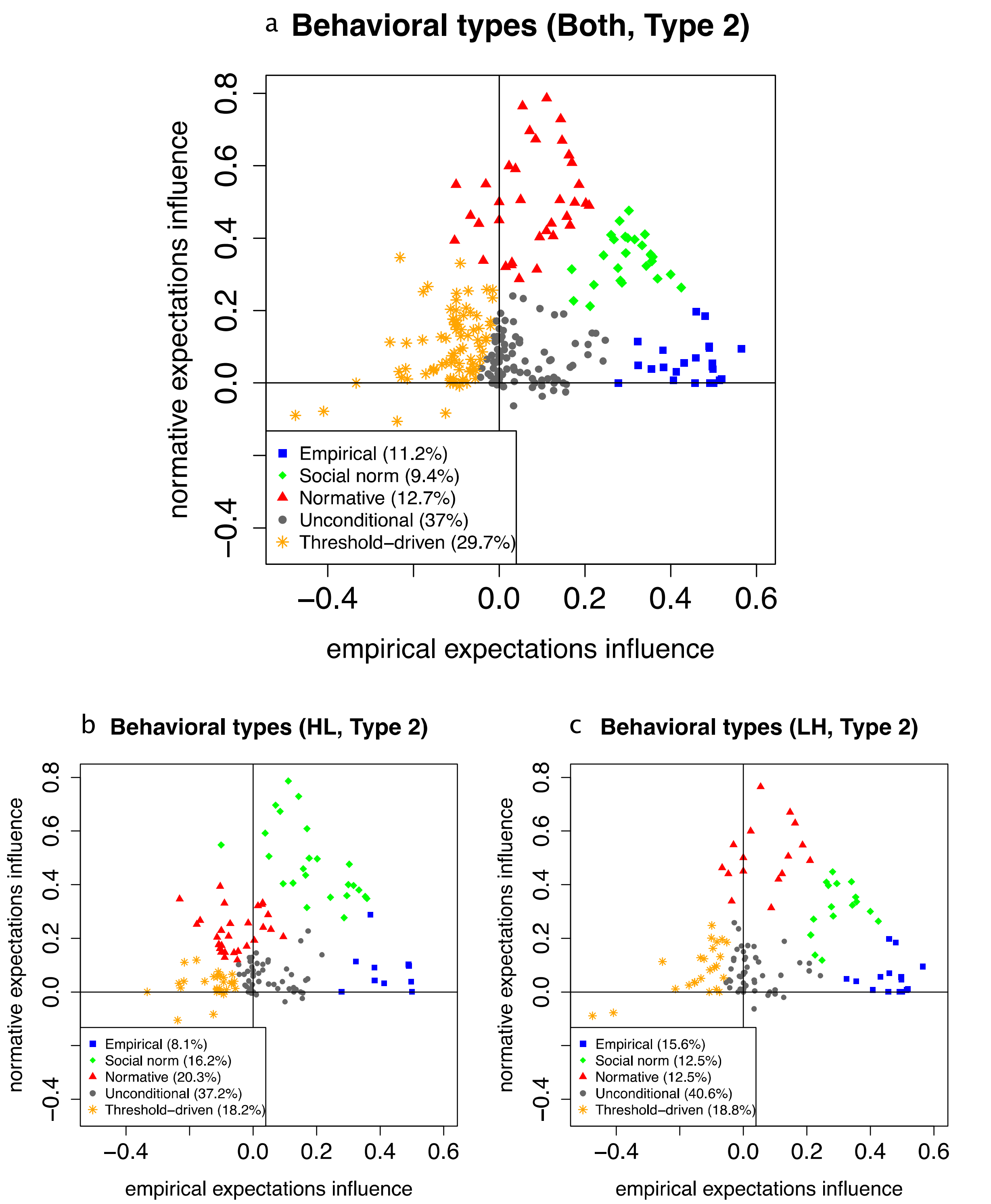

Supplementary Fig. 3. Behavioral types by treatments for Type 2. a empirical and normative expectations influence in both treatments combined. **b** empirical and normative expectations influence in the High Low treatment only. **c** empirical and normative expectations influence in the High Low treatment only. Source data are provided as a Source Data file.

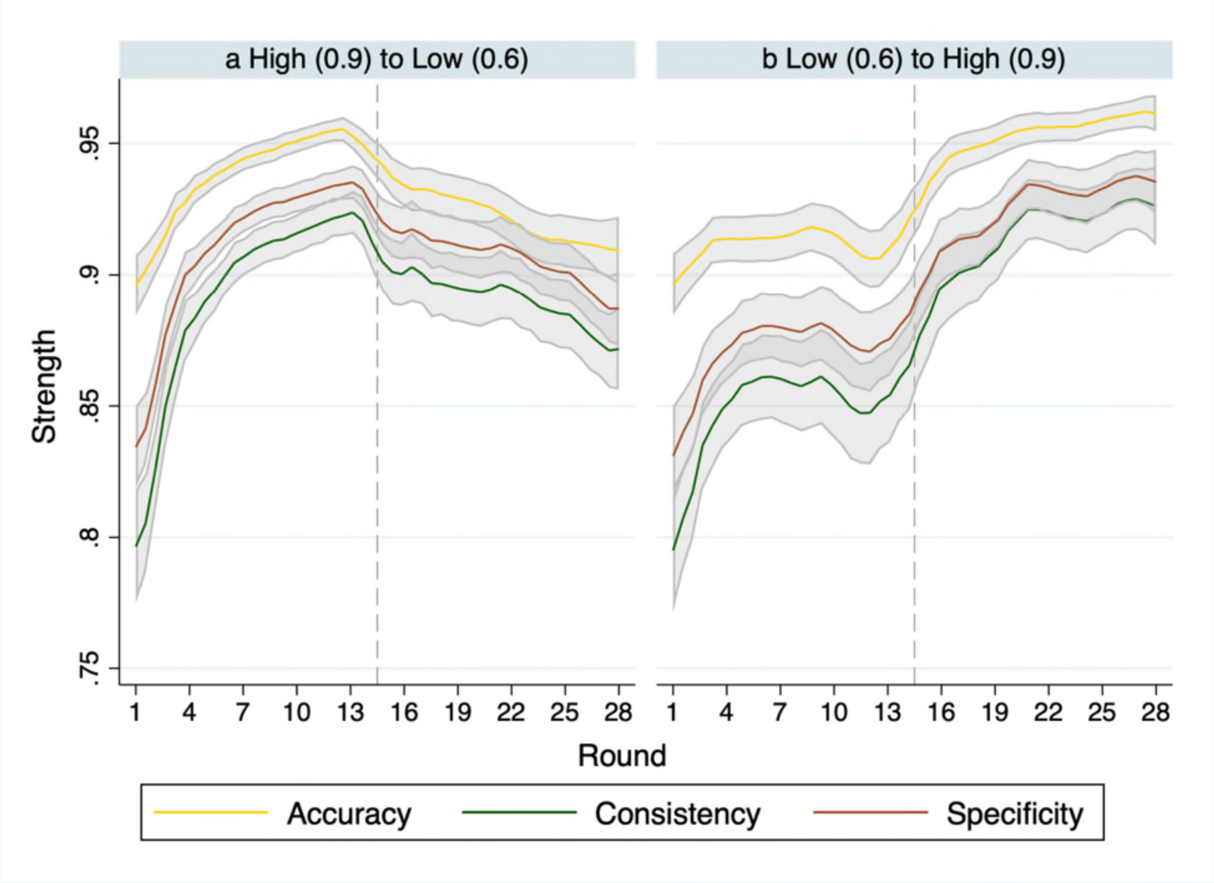

Supplementary Fig. 4. Social norm strength components for round and broken down by treatment. a High to Low treatment. **b** Low to High treatment. Means plotted. Shaded areas indicate 95% CIs with one observation per group. Source data are provided as a Source Data file.

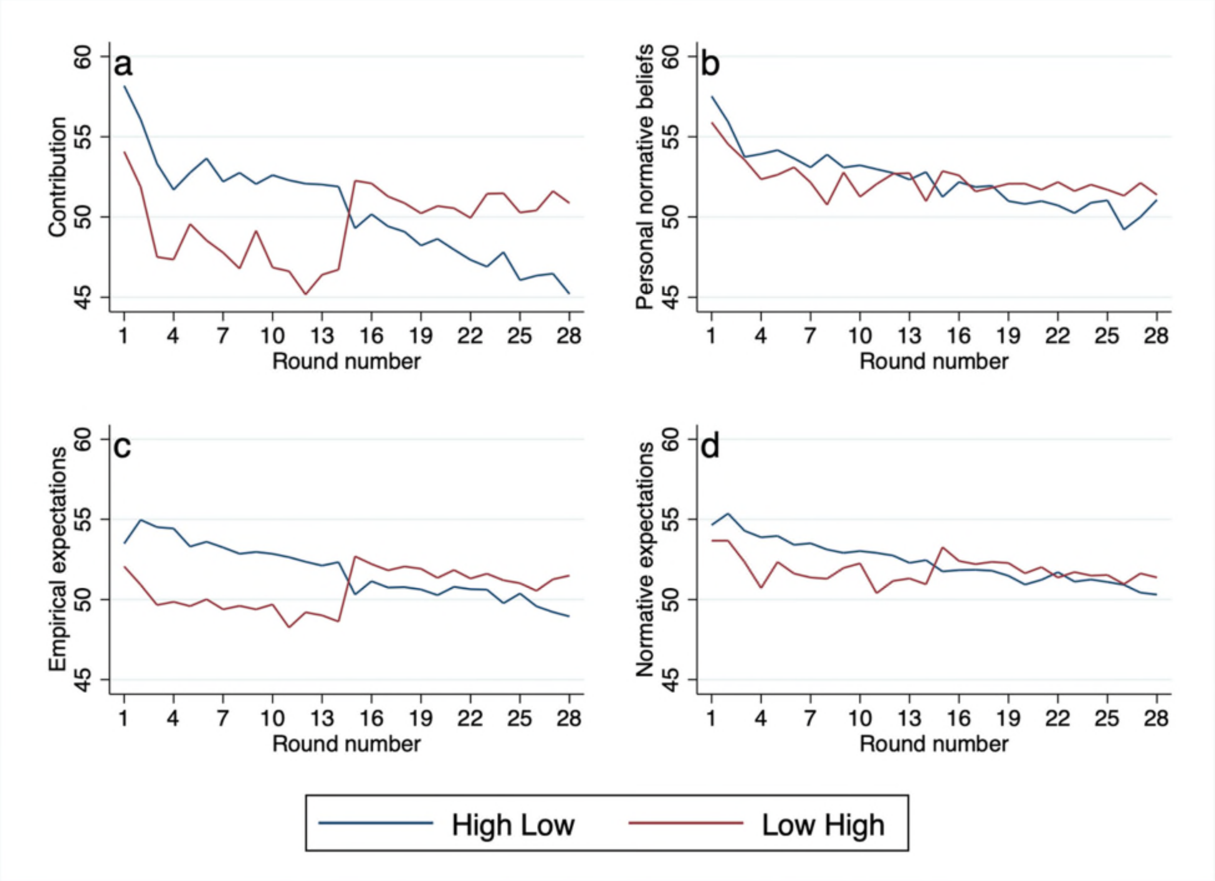

Supplementary Fig. 5. Contributions and expectations by round according to treatment. a Mean contribution by round. **b** Mean personal normative beliefs by round. **c** Mean empirical expectations by round. **d** Mean normative expectations by round. Source data are provided as a Source Data file.

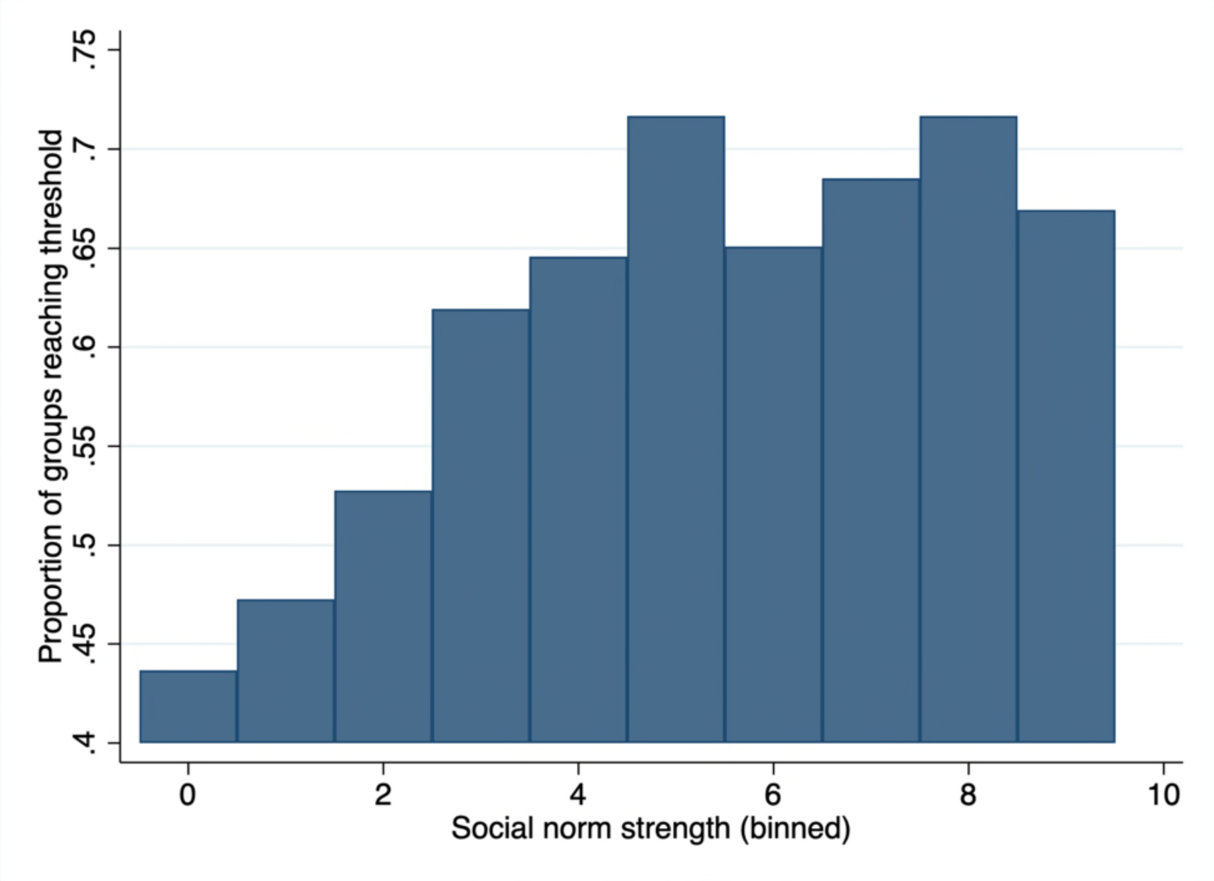

Supplementary Fig. 6. Proportion of groups reaching threshold according to social norm strength. Social norm strength grouped into ten nearly equal sets with either 126 or 127 group-level observations in each bin. 0 contains social norm strengths [.263, .591], 1 contains [.591, .656], 2 contains [.657, .704], 3 contains [.704, .741], 4 contains [.741, .772], 5 contains [.772, .802], 6 contains [.802, .828], 7 contains [.828, .852], 8 contains [.852, .877], 9 contains [.877, 1]. Groups with 3 or more inactive/excluded subjects are excluded. Source data are provided as a Source Data file.

Supplementary References

1. Bicchieri, C. *The Grammar of Society: The Nature and Dynamics of Social Norms*. (Cambridge University Press, 2006).
2. Konow, J. Fair shares: Accountability and cognitive dissonance in allocation decisions. *American Economic Review* **90**, 1072–1091 (2000).
3. Croson, R. & Konow, J. Social preferences and moral biases. *Journal of Economic Behavior & Organization* **69**, 201–212 (2009).
4. Cialdini, R., Reno, R. R. & Kallgren, C. A. A focus theory of normative conduct: Recycling the concept of norms to reduce littering in public places. *Journal of Personality and Social Psychology* **58**, 1015–1026 (1990).
5. Bicchieri, C. & Xiao, E. Do the right thing: but only if others do so. *J. Behav. Decis. Making* **22**, 191–208 (2009).
6. Bicchieri, C. Words and deeds: A focus theory of norms. in *Rationality, Rules, and Structure* (eds. Nida-Rümelin, J. & Spohn, W.) 153–184 (Springer Netherlands, 2000). doi:10.1007/978-94-015-9616-9_10.
7. Xiao, E. & Houser, D. Avoiding the sharp tongue: Anticipated written messages promote fair economic exchange. *Journal of Economic Psychology* **30**, 393–404 (2009).
8. Price, R. H. & Bouffard, D. L. Behavioral appropriateness and situational constraint as dimensions of social behavior. *Journal of Personality and Social Psychology* **30**, 579–586 (1974).
9. Gelfand, M. J. *et al.* Differences between tight and loose cultures: A 33-nation study. *Science* **332**, 1100–1104 (2011).
