# Supplementary material for: Evidence from a long-term experiment that collective risks change social norms and promote cooperation
Source: Nat Commun. 2021 Sep 15;12:5452. doi: 10.1038/s41467-021-25734-w (PMC8443614; doi:10.1038/s41467-021-25734-w)
Supplement: Supplementary file 3 — Reporting summary [file 41467_2021_25734_MOESM3_ESM.pdf]

nature research

Corresponding author(s): Aron Szekely

Last updated by author(s): Aug 4, 2021

# Reporting Summary

Nature Research wishes to improve the reproducibility of the work that we publish. This form provides structure for consistency and transparency in reporting. For further information on Nature Research policies, see our Editorial Policies and the Editorial Policy Checklist.

## Statistics

For all statistical analyses, confirm that the following items are present in the figure legend, table legend, main text, or Methods section.

| n/a | Confirmed | |
|---|---|---|
| ☐ | ☒ | The exact sample size (*n*) for each experimental group/condition, given as a discrete number and unit of measurement |
| ☐ | ☒ | A statement on whether measurements were taken from distinct samples or whether the same sample was measured repeatedly |
| ☐ | ☒ | The statistical test(s) used AND whether they are one- or two-sided<br>*Only common tests should be described solely by name; describe more complex techniques in the Methods section.* |
| ☐ | ☒ | A description of all covariates tested |
| ☐ | ☒ | A description of any assumptions or corrections, such as tests of normality and adjustment for multiple comparisons |
| ☐ | ☒ | A full description of the statistical parameters including central tendency (e.g. means) or other basic estimates (e.g. regression coefficient) AND variation (e.g. standard deviation) or associated estimates of uncertainty (e.g. confidence intervals) |
| ☐ | ☒ | For null hypothesis testing, the test statistic (e.g. *F*, *t*, *r*) with confidence intervals, effect sizes, degrees of freedom and *P* value noted<br>*Give P values as exact values whenever suitable.* |
| ☒ | ☐ | For Bayesian analysis, information on the choice of priors and Markov chain Monte Carlo settings |
| ☒ | ☐ | For hierarchical and complex designs, identification of the appropriate level for tests and full reporting of outcomes |
| ☒ | ☐ | Estimates of effect sizes (e.g. Cohen's *d*, Pearson's *r*), indicating how they were calculated |

*Our web collection on statistics for biologists contains articles on many of the points above.*

## Software and code

Policy information about availability of computer code

| | |
|---|---|
| Data collection | The experiments were conducted with oTree (IBSEN version) and participants were recruited through the IBSEN subject pool (http://www.ibsen-h2020.eu) |
| Data analysis | Data analysis is available at https://osf.io/wvgk9/. For the statistical analysis we used two softwares: STATA IC 16.1 for econometric analysis and R (3.6.3) for clustering analysis and power analysis (code available at https://osf.io/wvgk9/). Our preregistration is available at: https://osf.io/f3cyt. |

For manuscripts utilizing custom algorithms or software that are central to the research but not yet described in published literature, software must be made available to editors and reviewers. We strongly encourage code deposition in a community repository (e.g. GitHub). See the Nature Research guidelines for submitting code & software for further information.

## Data

Policy information about availability of data

All manuscripts must include a data availability statement. This statement should provide the following information, where applicable:

- Accession codes, unique identifiers, or web links for publicly available datasets
- A list of figures that have associated raw data
- A description of any restrictions on data availability

The data generated in this study have been deposited in the Open Science Framework database under accession code DOI 10.17605/OSF.IO/WVGK9 (https://osf.io/wvgk9/).

# Field-specific reporting

Please select the one below that is the best fit for your research. If you are not sure, read the appropriate sections before making your selection.

- [ ] Life sciences
- [x] Behavioural & social sciences
- [ ] Ecological, evolutionary & environmental sciences

For a reference copy of the document with all sections, see nature.com/documents/nr-reporting-summary-flat.pdf

# Behavioural & social sciences study design

All studies must disclose on these points even when the disclosure is negative.

Study description

We use a 30-day online experiment in which we monitor social norm evolution to test if the threat of a collective loss changes their strength and their effect on cooperative behavior. Subjects participate in 28 rounds of our version of the collective-risk social dilemma in groups of six, play one round per day, and the total experiment runs over 30 days. On day 1, subjects completed a range of measures/questionnaires: the Big Five, Social Value Orientation slider measure, Autism spectrum, and risk preference. On day 2 - 29 they participated in our version of the collective-risk social dilemma. On day 30, they participated in punishment preference elicitation, punishment expectations elicitation, and a final questionnaire,

In the 28 day cooperation setting, subjects need to cooperate at, or above, a threshold level to prevent the possibility of collective loss occurring. If the threshold is not reached then there is a probability that all subjects lose everything (the risk probability), but, there is also a probability that they keep what they did not contribute. Subjects have to decide how much of their endowment to contribute to the common pot (between 0 – 100). All contributions to the pot are destroyed. However, if a sufficient total amount is contributed, the risk of collective loss is averted with certainty.

We implement four treatments in a mixed within- and between-subjects design. The within-subjects treatments change the risk probability (0.9 or 0.6). Subjects face one risk probability for 14 rounds and in the other 14 rounds they face a different risk probability. The between-subjects treatments vary the ordering: whether subjects face a 0.9 risk and then a 0.6 risk or vice versa.

To study whether subjects' contribution was driven by the presence of social norms, we elicited subjects' Personal Normative Beliefs (PNB), Empirical Expectations (EE) and Normative Expectations (NE) every round. We also manipulated their EE and NE on selected rounds to understand their causal effect on contribution.

Research sample

We recruited and began the experiment with 300 subjects (150 in each treatment). We used the software program R to conduct a power analysis. In accordance with List (2011) our goal was to obtain at least a 0.80 power with a significance level of 0.05, we have $t_{\alpha/2} = 1.96$ and $t_{\beta} = 0.84$ from standard normal tables. Thus, to detect an effect size of three in the outcome variable one would need $n^* = 142$ observations in each treatment, hence a total amount of 284 participants.

Sampling strategy

Subjects were recruited from the IBSEN subject pool, are Spanish or resident in Spain, and have an average age of 26 years old, 49% of the subjects were female, 50% of them were students of the University of Carlos III of Madrid. We recruited 300 subjects in order to cover the risk of drop-out.

The strategy for protecting the experiment from the drop-out risk was the following. If dropout was less than 15%, (an upper bound estimates which comes from previous on-line experiments run by the IBSEN project: http://www.ibsen-h2020.eu), we would have stopped collecting the data after we run the experiment with 300 participants (which is what happened). If dropout was more than 15% (that is less than 255 participants remain at the end of the experiment) we would have run sessions to increase the number of subjects up to a maximum of 300. We would have recruited further subjects in proportion to the dropout according to the treatment and we would have run additional sessions so that we have approximately equal subjects in each session. We did not use the second strategy because dropout was sufficiently low.

Data collection

Participants were contacted through email and they played the online experiment either via computer or via mobile phone. The researchers were not physically present during the experiments and hence could not influence the participants' decision. The researcher was not blinded to experimental condition nor to study hypothesis, but this knowledge could not influence participant's behavior.

Timing

Data collection occurred in two sessions that lasted one month each: the High-Low session started on the 4th of June 2018 and the Low-High session started on the 3rd of September 2018. Both lasted 30 days.

Data exclusions

We exclude the decision of subjects who did not actively make them (i.e. either due to inactivity or exclusion) during the experiment. The total number of dropouts amounts to 23 subject overall (3 in the High Low session and 20 in the Low High session). The exclusion criteria were established by the time of the pre-registration (please see the corresponding section at https://osf.io/f3cyt)

Non-participation

We excluded subjects for two reasons:
1) if they missed the first day of decision
2) if they were inactive (i.e. they did not take any decision) for 3 times in total during the whole game.
If the subject was considered inactive, he/she was not be included in the analysis. We tested whether the dropout was not random by checking if the missing subjects were different from the non-missing ones within each treatment.

Randomization

Subjects were recruited through the IBSEN online platform (http://www.ibsen-h2020.eu) and they were randomly assigned to one of the two treatments, i.e. Treatment 1 and Treatment 2. We checked whether the randomization assumption were true by comparing

the average and distributions of the psychological predictors and questionnaire that we elicit on day 1. In Treatment 1, subjects started with a probability of disaster at 0.9 for the first 14 days (from 1 to 14) and then the probability of the disaster is recued to 0.6 for the remaining 14 days (from 15 to 28). Treatment 2 is exactly the reverse such that subjects start with a probability of disaster of 0.6 (from day 1 to 14) and then this changes to 0.9 from day 15 to 28.

# Reporting for specific materials, systems and methods

We require information from authors about some types of materials, experimental systems and methods used in many studies. Here, indicate whether each material, system or method listed is relevant to your study. If you are not sure if a list item applies to your research, read the appropriate section before selecting a response.

## Materials & experimental systems

| n/a | Involved in the study |
|---|---|
| ☒ | ☐ Antibodies |
| ☒ | ☐ Eukaryotic cell lines |
| ☒ | ☐ Palaeontology and archaeology |
| ☒ | ☐ Animals and other organisms |
| ☐ | ☒ Human research participants |
| ☒ | ☐ Clinical data |
| ☒ | ☐ Dual use research of concern |

## Methods

| n/a | Involved in the study |
|---|---|
| ☒ | ☐ ChIP-seq |
| ☒ | ☐ Flow cytometry |
| ☒ | ☐ MRI-based neuroimaging |

# Human research participants

Policy information about studies involving human research participants

| | |
|---|---|
| Population characteristics | See above. |
| Recruitment | See above. Subjects were recruited through the IBSEN online platform (http://www.ibsen-h2020.eu).<br>It is difficult to see how any self-selection bias could drive our main findings given that the participants are allocated randomly between the two treatments. |
| Ethics oversight | The study complied with all relevant ethical regulations for work with human participants such as obtaining informed consent. The study received institutional ethical approval from the Institute of Cognitive Sciences and Technologies (Italian National Research Council, Rome, Italy). |

Note that full information on the approval of the study protocol must also be provided in the manuscript.
